# Novel α-Aminophosphonates and α-Aminophosphonic Acids: Synthesis, Molecular Docking and Evaluation of Antifungal Activity against *Scedosporium* Species

**DOI:** 10.3390/molecules27123886

**Published:** 2022-06-17

**Authors:** Anthonny Cordero-Díaz, Efren Robledo-Leal, Eugenio Hernández-Fernández, Emanuel Hernández-Núñez, Mariana Elizondo-Zertuche, Susana T. López-Cortina

**Affiliations:** 1 Laboratorio de Química Industrial, Facultad de Ciencias Químicas, Universidad Autónoma de Nuevo León, Av. Universidad s/n Cd. Universitaria, San Nicolás de los Garza 66455, Nuevo León, Mexico; anthonny.corderodz@uanl.edu.mx (A.C.-D.); eugenio.hernandezfr@uanl.edu.mx (E.H.-F.); 2 Laboratorio de Micología y Fitopatología, Facultad de Ciencias Biológicas, Universidad Autónoma de Nuevo León, Av. Universidad s/n Cd. Universitaria, San Nicolás de los Garza 66455, Nuevo León, Mexico; efren.robledoll@uanl.edu.mx; 3 Departamento de Recursos del Mar, Centro de Investigación y de Estudios Avanzados del Instituto Politécnico Nacional, Unidad Mérida, Mérida 97310, Yucatán, Mexico; emanuel.hernandez@cinvestav.mx; 4 Departamento de Microbiología, Facultad de Medicina, Universidad Autónoma de Nuevo León, Madero y Dr. Aguirre Pequeño, Col. Mitras Centro, Monterrey 64460, Nuevo León, Mexico

**Keywords:** α-aminophosphonates, green chemistry, antifungal activity, genus *Scedosporium*

## Abstract

The *Scedosporium* genus is an emerging pathogen with worldwide prevalence and high mortality rates that gives multidrug resistance to antifungals; therefore, pharmacological alternatives must be sought for the treatment of diseases caused by this fungus. In the present project, six new α-aminophosphates were synthesized by the Kabachnik–Fields multicomponent reaction by vortex agitation, and six new monohydrolyzed α-aminophosphonic acids were synthesized by an alkaline hydrolysis reaction. Antifungal activity was evaluated using the agar diffusion method as an initial screening to determine the most active compound compared to voriconazole; then it was evaluated against 23 strains of the genus *Scedosporium* following the M38-A2 protocol from CLSI (activity range: 648.76–700 µg/mL). Results showed that compound **5f** exhibited the highest antifungal activity according to the agar diffusion method (≤1 mg/mL). Cytotoxicity against healthy COS-7 cells was also evaluated by the MTT assay and it was shown that compound **5f** exhibits a lower toxicity in comparison to voriconazole at the same concentration (1000 µM). A docking study was conducted afterwards, showing that the possible mechanism of action of the compound is through the inhibition of allosteric 14-α-demethylase. Taking these results as a basis, **5f** is presented as a compound with attractive properties for further studies.

## 1. Introduction

Invasive mycoses are responsible for almost 1.7 million deaths per year [1], this data is similar to the reported deaths caused by tuberculosis (1.4 million deaths per year) [2] and higher than the 409,000 cases reported for malaria [3]. However, public health programs in charge of monitoring fungal diseases are generally lacking in timely identification as well as treatment [4]; in fact, most public institutions around the world do not carry out mandatory surveillance of infections caused by fungi [5]. In addition, there is a concern about drug resistance (acquired or intrinsic) to available antifungals [6] and the advent of emerging fungal pathogens, such as *Scedosporium* species and *Lomentospora prolificans* [7]. 

*Scedosporium* species are emerging pathogens with worldwide prevalence and high mortality rates, especially in immunocompromised patients, with a broad spectrum of clinical manifestations from the least severe, such as colonization of the respiratory tract and superficial infections, to the most severe, such as invasive localized or disseminated mycoses [8]. *Scedosporium* species have been isolated in places subject to human activity, such as gardens, industrial parks, agricultural soils and polluted waters [9], these species are commonly multidrug-resistant, including fluconazole, amphotericin B, 5-fluorocytosine, anidulafungin, micafungin and caspofungin [10]. 

**Figure 1:**
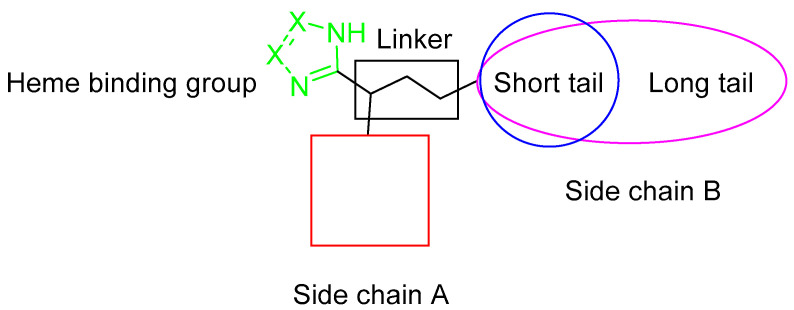
Structural modifications of azoles.

Focused on the treatment of infections caused by fungi, the modification of the structure of antifungal azoles is based on changes in the three rings and the size of the linker (Figure 1). The side chain A changes generally involve halogenated phenyl groups that occupied the hydrophobic pocket of lanosterol [11] and the side chain B modification is similar to the heme-binding group that helps improve pharmacokinetic properties [12]. On the other hand, the linker could be of various sizes, just like side chain B; some of these changes are ether, thioether, carbon (-CH_2_-, -CHR-, -CR_2_-), -NR_2_, -SO_2_-, heteroaromatic or heteroaliphatic rings, which are well-tolerated and have been considered good linkage types, while other groups such as amides, esters and -NHRs could reduce the antifungal activity [13,14,15]. There are many structural changes that have been made to improve antifungal activity [16]; for example, in the first generation of azoles, there is miconazole (IC_50_ = 0.057 µM *C. albicans*) [17], but when passing to the second generation with fluconazole, the activity is 5 times higher (IC_50_ = 0.010 µM *C. albicans*), changing 2,4-dichlorobenzene for 1,2,4-tetrazole, imidazole for 1,2,4-triazole and the chlorines of 2,4-dichlorobenzene for fluorine. On the other hand, changing the 1,2,4-triazole for 6-fluoro-2,4-pyrimidine and with the addition of a methyl group in the linker, voriconazole is found to have an activity level 5 times greater than its predecessor (IC_50_ = 0.002 µM *C. albicans*) [18,19]; despite these structural modifications, it is still necessary to search for alternative drugs that have a better therapeutic effect and fewer side effects, especially against the *Scedosporium* genus, since it has shown great resistance to azole antifungals. Thus, there is an important need to develop pharmacological alternatives for the treatment of infections caused by these and other type of fungi [20], so a promising route is the development of novel α-aminophosphonates and α-aminophosphonic monohydrolyzed acids, since these are structural analogues of α-amino acids and there have been previous reports regarding their broad biological activity, such as antiviral [21], anticancer [22], antibacterial [23] and antifungal functions [24]. The α-aminophosphonates could be obtained by the Kabachnik–Fields reaction, which is a one-pot multicomponent reaction between an amine, an aldehyde and a phosphite; there are many reports of conventional synthetic methods and non-conventional ones, such as microwave irradiation (MW) [25], with some of them involving the use of diverse catalysts [26], but there are virtually no reports regarding the synthesis of these compounds assisted by a vortex mixer with no catalysts needed, which constitutes the proposal of the present work.

On the other hand, α-aminophosphonic monohydrolyzed acids have not received much attention and they have been poorly reported, not only regarding their synthesis but also their biological activity. This is probably since in most of the reports, the efforts were directed to the total hydrolysis of diphosphonoester group [27,28], so the partial hydrolysis, as proposed here, represents an area of opportunity to further the development of organophosphorus compounds. Therefore, we describe noticeable results for the synthesis of novel α-aminophosphonates assisted by vortex mixer and the partial hydrolysis of the α-aminophosphonates by MW irradiation to obtain the corresponding monohydrolyzed compounds, following the principles of green chemistry in both methodologies.

## 2. Results and Discussion

### 2.1. Chemistry

**Scheme 1:**
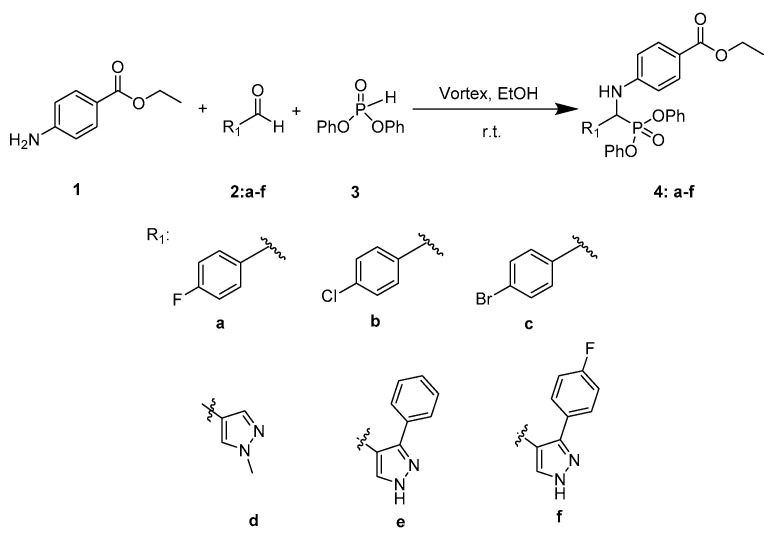
Synthesis of compounds **4a**–**f** by Kabachnik–Fields reaction.

First, the synthesis of six α-aminophosphonates was carried out according to the one pot Kabachnik–Fields reaction, in which the corresponding aldehyde (**2a**–**f**), ethyl 4-aminobenzoate (**1**) and diphenyl phosphite (**3**) were reacted in 4 mL of ethanol and stirred by a vortex mixer at room temperature (Scheme 1). For each compound, a white precipitate was observed at different reaction times and the yields were good to excellent (Table 1).

The synthesis of α-aminosphosphonates has been widely reported, including synthetic methodologies using ionic liquids [29], metallic salts as catalysts, such as Yb(OTf)_3_ [30] and HfCl_4_ [31], catalyst-free using aqueous ethyl lactate [32] among others; however, it is still necessary to find new and more sustainable methods for synthesis. Because of this, we hereby describe the synthesis of six new compounds performed using a vortex stirrer. This methodology showed some advantages over the typical laboratory round flask synthesis on a magnetic stirring hot plate, since vortex mixing provides a wide contact surface, high cutting speeds, greater mass transfer efficiency and micro-mixing regions [33], which are conditions that favor a decrease in the reaction time with good to excellent yields. Another advantage of the proposed methodology is the use of ethanol as a solvent due to its low toxicity and because it is a very common solvent in organic chemistry laboratories. Furthermore, all the products precipitated in this solvent are partially or totally insoluble in ethanol so they can be recovered by simple vacuum filtration; the partially soluble compounds were recovered by recrystallization from the same solvent to be subsequently filtered once it had been corroborated by thin-layer chromatography (TLC) that there was no longer any desired product in the mother liquor. In addition, most of the used solvent was recovered by distillation at reduced pressure for later reuse.

It is noteworthy that we found a trend concerning the percentage yields for halogenated compounds **4a**, **4b** and **4c**, in which as electronegativity increased and halogen size decreased, an increase in the yield was observed. This could suggest a significant effect of the halogen atom on the imine formation step, in which ethyl 4-aminobenzoate, **1**, reacts with the corresponding aldehyde (**2a**, **2b** or **2c**), supporting the greater electron-withdrawing effect of the fluor atom in the para position, which increases the electropositive character of the carbonyl carbon atom, favoring the imine formation.

Moreover, when the synthesis of compounds **4d**, **4e** and **4f** was performed using pyrazole aldehydes (**2d**, **2e**, **2f**), there was no significant difference on the reaction yields among them, but if they are compared globally, these yields are higher than they are when using halogenated aldehydes (compounds **4a**, **4b**, **4c**), suggesting an important electron-withdrawing effect of the pyrazole ring in the imine formation step.

**Scheme 2:**
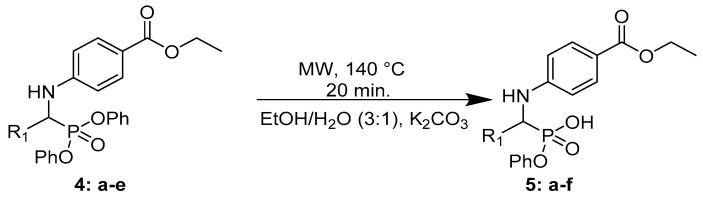
Synthesis of compounds **5a**–**f** by hydrolysis of **4a**–**f**.

The synthesis of six α-aminophosphonic monohydrolyzed acids was carried out by a basic hydrolysis between the previously obtained α-aminophosphonates (**4a**–**f**) and potassium carbonate under microwave irradiation at 140 °C for 20 min (Scheme 2). The reaction yields were moderate to good.

The methodology using microwave irradiation presented some advantages such as short reaction time (20 min), low amount of solvent (4 mL of ethanol/water 3:1) and a uniform heating process that improved the reaction performance.

Partial hydrolysis of α-aminophosphonates by conventional methodologies has been reported following harsh reaction conditions, e.g., by using nucleophilic bases, such as NaOH [34] or TMSBr [28], an expensive and corrosive reagent; therefore, the use of potassium carbonate as a catalyst is another advantage of our methodology because it is a weak base that can easily be recovered after the reaction by filtration and reused in subsequent reactions.

**Scheme 3:**
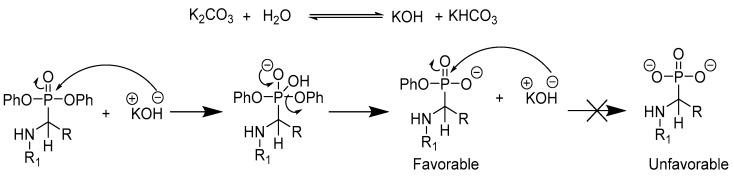
Putative reaction mechanism of α-aminophosphonate hydrolysis.

It is remarkable that the presence of water in the solvent mix helps potassium carbonate to yield a stronger potassium base in situ that can attack phosphorus and carry out the hydrolysis. Under these reaction conditions, partial hydrolysis of the phosphonate is observed and there was no evidence of total hydrolysis. The explanation for this could be that when the first phenoxy group is displaced, a negative charge on the oxygen bonded to the phosphorus atom is obtained; In the case of a second phenoxy group displacement, the phosphorous atom would have to bear two oxygens, both with negative charges (Scheme 3), which is not a favorable situation [35]. Additionally, phenylphosphite, ethyl 4-aminobenzoate and the corresponding aldehyde were observed as subproducts by TLC, suggesting a hydroxyl attack on the α-carbon.

The structure of the synthesized compounds was corroborated through the analysis of nuclear magnetic resonance (^31^P, ^1^H and ^13^C-NMR) spectra and high-resolution mass spectrometry (HRMS). In ^1^H-NMR, all the compounds (**4a**–**f** and **5a**–**f**) show the characteristic signals of the aliphatic protons corresponding to the ethoxy group (triplet and quartet). In addition, between 4.50 and 5.88 ppm, it was possible to observe the doublet corresponding to the *J* coupling CH-P, showing values from 19.9 to 25.4Hz. In ^13^C-NMR, *J* couplings C-P were observed for each compound with values between 139.2 and 165.1 Hz at chemical shifts ranging from 45.5–56.9 ppm. It is important to notice that for compounds **4a**, **4f**, **5a** and **5f**, it was possible to observe the large *J* coupling C-F with values of 244.3 and 248.1 Hz for **4a** and **4f** and 240.3 and 246.5 Hz for **5a** and **5f**, respectively (Table 2).

### 2.2. In Vitro Assays

The 12 synthesized compounds were evaluated by the agar diffusion method against the strains *S. boydii* (ZS-025), *S. apiospermum* (00-320) and *S. aurantiacum* (MC-070). Voriconazole was used as a control for the comparison of the inhibition halos of the compounds.; the inhibition halo readings were made at 72 h. The results showed that the series of α-aminophosphonates (**4a**–**f**) did not present measurable inhibition halos (i.e., ≥1 mm) at the different test concentrations; on the other hand, the series of monohydrolyzed α-aminophosphonic acids (**5a**–**f**) showed measurable inhibition halos. This might be due to the presence of the hydroxyl group that gives an acidic character to the molecules, which facilitates their diffusion through the medium, in contrast with the di-phosphonoesters of the α-aminophosphonate series where the interactions by hydrogen bonds with the medium are less significant, hindering proper diffusion [37]. From the series of acids, compound **5a** and **5f** resulted in inhibition halos comparable to voriconazole. Compound **5a** presented inhibition halos like voriconazole (10 mm ± 1 mm) at a concentration of 5 mg/mL. On the other hand, compound **5f** showed inhibition halos greater than those of voriconazole even at the lowest concentration evaluated (1 mg/mL) (Table 3). With these results, a second preliminary test was carried out with compound **5f**, with the aim of optimizing the evaluation of the susceptibility of 23 strains of the *Scedosporium* species against this compound according to the M38-A2 protocol, and the results showed a 100% inhibition of growth in the three evaluated species in the 500–700 µg/mL range. 

**Figure 2:**
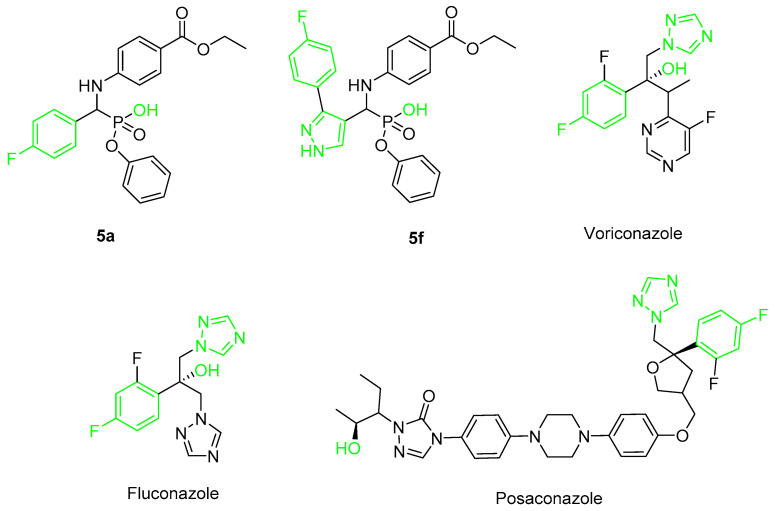
Structures of **5a**, **5f** and common antifungal azoles for comparison.

In relation to the analysis of the structure–activity relationship of the compounds with antifungal activity (**5a** and **5f**), leaving aside the presence of benzoate and phosphorous groups, which are common structural parts in both compounds, the presence of a fluorine atom in the para position of a phenyl ring is also common to both compounds; however, the presence of an azole type five-membered ring, such as pyrazole, enhances the activity of compound **5f**. This could be directly related to the structure of voriconazole (VRC) and other azoles, such as fluconazole (FLC) and posaconazole (PSC), which have these type of substituents in their structure [16] (Figure 2). Besides, it seems that the presence of a hydroxyl group could influence their diffusion through the medium and also their antifungal activity, since this group could interact with the glycans, chitins and glycoproteins present in the cell wall of most fungi [38], thus facilitating their diffusion into the cytoplasm. Therefore, the results obtained in this assay and the ones obtained by molecular docking study (Section 3.4) highlight the importance of including these moieties in the design of molecules with potential antifungal activity; also, in the specific case of compound **5f**, a synergistic effect of a pyrazole group with a fluorine atom and a hydroxyl group bonded to phosphorous atom of the aminophosphonic acid could be suggested.

### 2.3. Antifungal Activity Using the M38-A2 Protocol

The most active compound (**5f**) was evaluated against 23 strains of the *Scedosporium* species (five strains of *S. apiospermum*, *S. boydii*, *S. aurantiacum*, *S. dehoogii* respectively and three strains of *S. angustum*). The test range was 500–700 µg/mL. MIC_100_ was determined by geometric mean for compound **5f** and antifungals AMB, VRC and FLC; these values are shown in Table 4. The results showed accordance with those previously reported by Elizondo-Zertuche [10], where strains exhibited resistance to AMB with a geometric mean range of 6.96–16 µg/mL, FLC with a geometric mean range of 36.75–64 µg/mL and VRC with a geometric mean range of 0.44–2.64 µg/mL (Table 4). Regarding compound **5f**, this work represents the first study of a series of α-aminophosphonates and a series of monohydrolyzed α-aminophosphonic acids against different strains of *Scedosporium* species, so there is no report with which we can directly compare this study; however, an indirect comparison can be made with the publication by Mubarak [29] where the synthesis of a series of α-aminophosphonates was carried out and their activity was evaluated against strains of *Candida albicans*, *Fusarium oxysporum*, *Aspergillus flavus*, *A. niger* and *Cryptococcus neoformans*, reporting compounds that showed a MIC of 25 µg/mL, while FLC had a MIC of 6.25 µg/mL; although compound **5f** shows an activity range of 648.76–700 µg/mL, it is important to consider that *Scedosporium* species exhibit a greater resistance to FLC than other fungi, with values sometimes higher than 64 µg/mL (*S. aurantiacum* and *S. dehoogii*). Although the in vitro antifungal activity is low compared to VRC, compound **5f** exhibits lower cytotoxicity (Section 3.3), making it a viable and promising option for the treatment of infections caused by filamentous fungi that show antifungal resistance.

### 2.4. Cytotoxicity

To evaluate the cytotoxicity of the two compounds that showed activity by the agar diffusion method against the *Scedosporium* strains, the MTT test was performed in COS-7 cells at a concentration of 1000 µM of compounds **5a**, **5f** and VRC. This value is close to 700 µg for the compounds, which is the highest concentration tested by the CLSI protocol M38-A2; the reason for this was to be able to compare the toxicity of the compounds with VRC at high concentrations, and thus, to determine if, even with the high concentration of compound **5f** against *Scedosporium*, it does not become as toxic as the commercial drug (VRC). This test was also carried out for compound **5a** (Table 5). According to these results, it can be observed that despite the high MIC of compound **5f**, it has a lower toxicity than VRC. This is relevant because one of the main adverse effects of VRC is its hepatotoxicity [39], adding to the hypothesis of a possible mechanism of action for compound **5f** not involving a covalent union with the heme group (Section 3.4). This highlights the importance of further studies to verify its therapeutic efficacy using in vivo models.

### 2.5. Molecular Docking

Lanosterol-14α-demethylase is a key protein for ergosterol biosynthesis, a very important component of the fungal membrane responsible for its integrity and function. Membrane alterations cause pores, triggering cell death by osmotic pressure, so this protein represents the molecular target of many antifungals [40]. 

According to the results obtained by the in vitro tests of compounds **5a** and **5f**, it can be suggested that their mechanism of action is the inhibition of ergosterol synthesis by disruption of lanosterol-14α-demethylase. In order to evaluate this hypothesis, a molecular docking study was carried out. 

**Figure 3:**
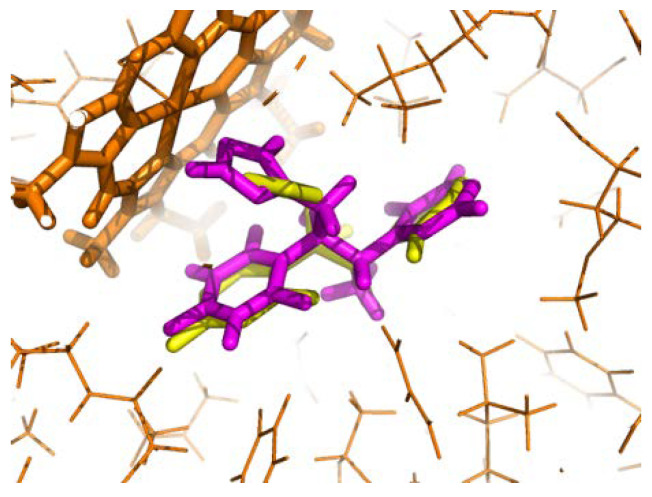
Ligand-binding site of lanosterol-14α-demethylase with co-crystallized native voriconazole (pink) and posed voriconazole as calculated by the AutoDock Vina program (yellow).

The active site of lanosterol-14α-demethylase (PDB ID: 5HS1) was validated with the co-crystallized native ligand, voriconazole. The spatial difference between the calculated and native ligand represented in the mean square deviation (RMSD) was 2.5 Å, indicating an adequate optimization for the coupling values (Figure 3). The validation was done with 1000 modes, ten replicates for each, selecting the lowest energy value. Protein visualization and overlap were carried out by using PyMOL 3.1 (Schrödinger, San Diego, CA, USA; http://www.pymol.org/; accessed on 16 May 2021).

Docking was carried out with the AutoDock Vina software (Scripps Research) using an automated protocol, which was previously validated with the prediction of binding between the protein and the crystal structure of voriconazole. The software successfully predicted the binding mode of the crystallized ligand with an RMSD of 2.5 Å.

**Figure 4:**
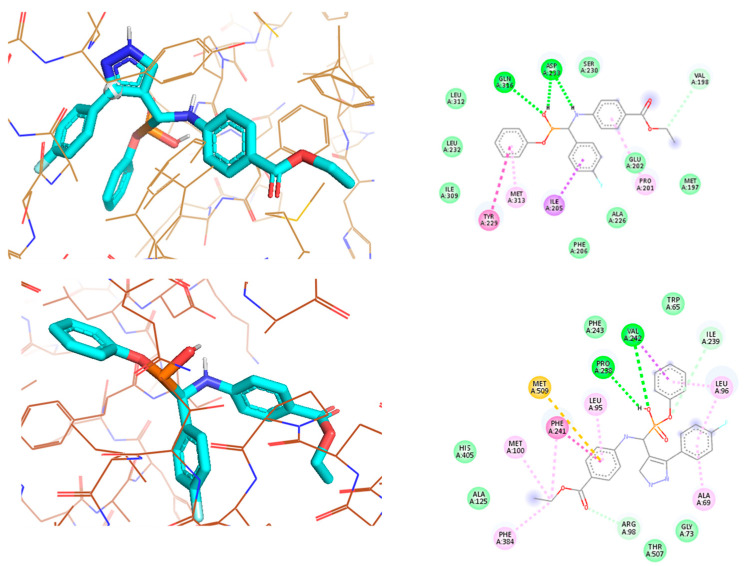
Interactions between compounds **5a**, **5f** and lanosterol-14α-demethylase.

According to the Discovery Studio Visualizer software, the interactions are observed between compound **5a** with the protein in its most favored pose and the hydrophobic pocket, formed by Leu312, Ser230, Met197, Glu202, Ala226, Phe206, Ile309 and Leu232. In addition to interactions between the hydrogen bond and the Gln316 and Asp233 residues, the aromatic rings exhibit interactions of the π type with Ile229 and Tyr229 (Figure 4). The compound **5f** is placed in the hydrophobic pocket formed by Ala125, His405, Phe243, Trp65, Ile239, Leu96, Gly73 and Thr507, and also exhibits a hydrogen bridge-type interaction with the Pro238 and Val242 residues and π type interactions between aromatic rings and Phe241, Met509, Leu95, Val242, Leu96 and Ala69 residues (Figure 4).

The results obtained by molecular docking show the importance of the substituents mentioned in the in vitro results section and suggest that the mechanism of action of the compounds is by allosteric inhibition, since there is no interaction of the molecules with the active site of the molecule. This could be associated with a reduction in the toxicity of the molecules **5a** and **5f** since the mechanism of action is not related to the covalent coordination of the heme group of lanosterol-14α-demethylase, as is the case for many antifungal azoles [41]. These data is supported by cytotoxic tests carried out in the previous section (Section 3.3). One of the major issues in the treatment of fungal diseases is the limited availability of compounds that possess good in vitro and in vivo antifungal activity with low cytotoxicity; these results show that compounds **5a** and **5f** are good candidates for further investigation of their therapeutic efficacy in vivo.

## 3. Materials and Methods

### 3.1. Synthesis of α-Aminophosphonates

Ethyl 4-aminobenzoate (1 eq.), the corresponding aldehyde (1 eq.) and diphenyl phosphite (1 eq.) were added to a sealed glass tube and stirred by a vortex mixer (Vortex-Genie model G205) until a precipitate was observed at different corresponding reaction times. The precipitate was filtered under vacuum and washed with ethanol, the mother liquor was recollected, concentrated and cooled for recrystallization. 

### 3.2. Synthesis of Monohydrolyzed α-Aminophosphonates 

Previously obtained α-Aminophosphonate (1 eq.), potassium carbonate (1 eq.) and 4 mL of ethanol/water (3:1) were added to a G10 microwave tube with a magnetic stirrer. The mixture was exposed to microwave irradiation (microwave synthesis reactor Monowave 300 Anton-Paar) at 140 °C for 20 min and monitored by TLC. The reaction crude was purified by column chromatography using silica gel as the stationary phase and methanol/ethyl acetate (7:3) as an eluent. 

#### 3.2.1. Ethyl-4-(((diphenoxyphosphoryl)(4-fluorophenyl)methyl)amino)benzoate, **4a**

White powder (1.03 g, 77%).^1^H NMR (400 MHz, DMSO_-_*d*_6_) δ(ppm): 1.25 (t, *J* = 7.1 Hz, 3H, **CH_3_**CH_2_O), 4.20 (q, *J* = 7.1 Hz, 2H, CH_3_**CH_2_**O), 5.86 (dd, *J* = 24.9, 10.2 Hz, 1H, P**-CH-**NH), 6.91 (d, *J* = 8.7 Hz, 2H, H_arom_), 6.98–7.07 (m, 4H, H_arom_), 7.14–7.21 (m, 2H, H_arom_), 7.23–7.27 (m, 2H, H_arom_), 7.30–7.36 (m, 4H, H_arom_), 7.65 (dd, *J* = 10.2, 5.1 Hz, 1H, CH-**NH**-P), 7.71 (d, *J* = 8.9 Hz, 2H, H_arom_), 7.75–7.79 (m, 2H, H_arom_). ^13^C NMR (100 MHz, DMSO-*d_6_*) δ(ppm): 14.3, 53.1 (d, *J*_CP_ = 156.2 Hz), 59.7, 112.7, 115.3 (d, *J =* 21.6 Hz), 118.2, 120.3 (d, *J =* 4.0 Hz), 120.5 (d, *J =* 4.0 Hz), 125.3 (d, *J =* 2.9 Hz), 129.8 (d, *J =* 4.4 Hz), 130.7(d, *J =* 14.6 Hz), 131.3 (d, *J =* 2.9 Hz), 149.9 (d, *J =* 10.0 Hz), 150.0 (d, *J =* 10.0 Hz), 151.2 (d, *J =* 12.4 Hz), 161.9 (d, *J_CF_* = 244.3 Hz), 163.1, 165.7 (C=O). ^31^P NMR (162 MHz, DMSO-*d_6_*): δ(ppm): 15.58. HRMS (ESI^+^) m/z calcd. for C_28_H_26_FNO_5_P [M + H]^+^ 506.1533; found 506.1464. 

#### 3.2.2. Ethyl-4-(((4-chlorophenyl)(diphenoxyphosphoryl)methyl)amino)benzoate, **4b**

White powder (1.55 g, 65%). ^1^H NMR (400 MHz, DMSO_-_*d*_6_) δ(ppm): 1.25 (t, *J* = 7.1 Hz, 3H, **CH_3_**CH_2_O), 4.20 (q, *J* = 7.1 Hz, 2H, CH_3_**CH_2_**O), 5.88 (dd, *J* = 25.1, 10.3 Hz, 1H, P**-CH-**NH), 6.96 (d, *J* = 8.3 Hz, 2H, H_arom_), 6.99–7.07 (m, 4H, H_arom_), 7.14–7.20 (m, 2H, H_arom_), 7.29–7.37 (m, 4H, H_arom_), 7.48 (d, *J* = 8.3 Hz, 2H, H_arom_), 7.67 (dd, *J* = 10.3, 5.4 Hz, 1H, CH-**NH**-P), 7.69–7.78 (m, 4H, H_arom_). ^13^C NMR (100 MHz, DMSO-*d_6_*) δ(ppm): 14.3, 53.2 (d, *J*_CP_ =156.2 Hz), 59.7, 112.7, 118.3, 120.3 (d, *J =* 4.0 Hz), 120.5 (d, *J =* 3.9 Hz), 125.3, 128.4 (d, *J =* 2.2 Hz), 129.8 (d, *J =* 6.7 Hz), 130.4 (d, *J =* 5.9 Hz), 130.7, 132.9 (d, *J =* 3.8 Hz), 134.3, 149.8 (d, *J =* 10.0 Hz), 150.0 (d, *J =* 10.0 Hz), 151.1 (d, *J =* 12.5 Hz), 165.7 (C=O). ^31^P NMR (162 MHz, DMSO-*d_6_*): δ(ppm): 15.24. HRMS (ESI^+^) *m*/*z* calcd. for C_28_H_26_ClNO_5_P [M + H]^+^ 522.1159; found 522.1128.

#### 3.2.3. Ethyl-4-(((4 bromophenyl)(diphenoxyphosphoryl)methyl)amino)benzoate, **4c**

White powder (1.17 g, 50%). ^1^H NMR (400 MHz, DMSO_-_*d*_6_) δ(ppm): 1.25 (t, *J* = 7.1 Hz, 3H, **CH_3_**CH_2_O), 4.20 (q, *J* = 7.1 Hz, 2H, CH_3_**CH_2_**O), 5.86 (dd, *J* = 25.4, 10.2 Hz, 1H, P**-CH-**NH), 6.95 (d, *J* = 8.1 Hz, 2H, H_arom_), 6.99–7.04 (m, 4H, H_arom_), 7.16–7.20 (m, 2H, H_arom_), 7.31–7.36 (m, 4H, H_arom_), 7.58–7.71 (m, 7H, H_arom_ and **NH**). ^13^C NMR (100 MHz, DMSO-*d_6_*) δ(ppm): 14.3, 53.2 (d, *J*_CP_ = 156.4 Hz), 59.8, 112.7, 118.2, 120.3 (d, *J =* 4.0 Hz), 120.5 (d, *J =* 4.0 Hz) 121.5 (d, *J =* 4.0 Hz), 125.4, 129.9 (d, *J =* 7.3 Hz), 130.7, 130.8 (d, *J =* 6.0 Hz) 131.6 (d, *J =* 2.4 Hz), 134.7, 149.8 (d, *J =* 10.0 Hz), 150.0 (d, *J =* 10.0 Hz) 151.2 (d, *J =* 12.6 Hz), 165.7 (C=O). ^31^P NMR (162 MHz, DMSO_-_*d*_6_) δ(ppm): 15.07. HRMS (ESI^+^) *m*/*z* calcd. for C_28_H_26_BrNO_5_P [M + H]^+^ 566.0732; found 566.0699.

#### 3.2.4. Ethyl-4-(((diphenoxyphosphoryl)(1-methyl-1H-pyrazol-4-yl)methyl)amino)benzoate, **4d**

White powder (0.84 g, 92%). ^1^H NMR (400 MHz, CDCl_3_) δ(ppm): 1.35 (t, *J* = 7.1 Hz, 3H, **CH_3_**CH_2_O), 3.74 (s, 3H, N-**CH_3_**), 4.31 (q, *J* = 7.1 Hz, 2H, CH_3_**CH_2_**O), 5.25 (d, *J* = 22.1 Hz, 1H, **CH**-P), 6.66 (d, *J* = 8.8 Hz, 2H, H_arom_), 6.95–7.32 (m, 10H, H_arom_ and **NH**), 7.46–7.51 (m, 1H, H_arom_), 7.60 (s, 1H, H_arom_), 7.85 (d, *J* = 8.7 Hz, 2H, H_arom_). These data are consistent with the literature [36].

#### 3.2.5. Ethyl-4-(((diphenoxyphosphoryl)(3-phenyl-1H-pyrazol-4-yl)methyl)amino)benzoate, **4e**

White powder (1.47 g, 98%).^1^H NMR (400 MHz, DMSO-*d_6_*) δ(ppm): 1.25 (t, *J* = 7.1 Hz, 3H, **CH_3_**CH_2_O), 4.19 (q, *J* = 7.1 Hz, 2H, CH_3_**CH_2_**O), 5.39 (dd, *J* = 18.8, 9.4 Hz,1H, P**-CH-**NH), 6.73 (d, *J* = 8.5 Hz, 2H, H_arom_), 6.89 (d, *J* = 8.0 Hz, 2H, H_arom_), 7.02 (d, *J* = 8.1 Hz, 2H, H_arom_), 7.15–7.18 (m, 2H, H_arom_), 7.28–7.34 (m, 4H, H_arom_), 7.39–7.46 (m, 4H, H_arom_ and **NH**), 7.53–7.55 (m, 2H, H_arom_), 7.66 (d, *J* = 8.5 Hz, 2H, H_arom_), 8.03 (s, 1H, H_arom_), 13.23 (bs, 1H, **NH**). ^13^C NMR (100 MHz, DMSO-*d_6_*) δ(ppm): 14.3, 45.5 (d, *J*_CP_ = 165.1 Hz), 59.8, 111.1 (d, *J =* 2.3 Hz), 112.1, 118.0, 120.2 (d, *J =* 3.9 Hz), 120.4 (d, *J =* 3.9 Hz), 125.4, 127.9, 128.9, 129.9 (d, *J =* 4.3 Hz), 130.8, 149.9 (d *J =* 10.3 Hz), 150.0 (d *J =* 10.3 Hz), 150.7 (d, *J =* 7.0 Hz), 165.7 (C=O). ^31^P NMR (162 MHz, DMSO_-_*d*_6_) δ(ppm): 16.00. HRMS (ESI^+^) *m*/*z*, calcd. for C_31_H_29_N_3_O_5_P [M + H]^+^ 554.1845; found 554.1801.

#### 3.2.6. Ethyl-4-(((diphenoxyphosphoryl)(3-(4-fluorophenyl)-1H-pyrazol-4-yl)methyl)amino)benzoate, **4f**

White powder (1.36 g, 95%).^1^H NMR (400 MHz, DMSO-*d_6_*) δ(ppm): 1.25 (t, *J* = 7.1 Hz, 3H, **CH_3_**CH_2_O), 4.20 (q, *J* = 7.1 Hz, 2H, CH_3_**CH_2_**O), 5.39 (dd, *J* = 19.9, 9.9 Hz, 1H, P**-CH-**NH), 6.75 (d, *J* = 8.4 Hz, 2H, H_arom_), 6.91 (d, *J* = 8.0 Hz, 2H, H_arom_), 7.02 (d, *J* = 8.0 Hz, 2H, H_arom_), 7.15–7.19 (m, 2H, H_arom_), 7.28–7.37 (m, 7H, H_arom_ and **NH**), 7.57–7.60 (m, 2H, H_arom_), 7.67 (d, *J* = 8.4 Hz, 2H, H_arom_), 8.08 (bs, 1H, H_arom_), 13.18 (bs, 1H, **NH**). ^13^C NMR (100 MHz, DMSO-*d_6_*) δ(ppm): 14.3, 45.5 (d, *J*_CP_ = 164.7 Hz), 59.8, 111.1 (d, *J =* 2.3 Hz), 112.1, 115.8, 118.0, 120.2 (d, *J =* 3.9 Hz), 120.4 (d, *J =* 3.9 Hz), 125.4 (d, *J =* 3.7 Hz), 129.9 (d, *J =* 6.3 Hz), 130.0, 130.8, 149.9 (d, *J =* 10.3 Hz), 150.0 (d, *J =* 10.7 Hz) 150.7 (d, *J =* 7.1 Hz), 163.2 (d, *J*_CF_ =248.1 Hz) 165.7 (C=O). ^31^P NMR (162 MHz, DMSO_-_*d*_6_) δ(ppm): 15.97. HRMS (ESI^+^) *m*/*z*, calcd. for C_31_H_28_FN_3_O_5_P [M + H]^+^ 572.1751; found 572.1630.

#### 3.2.7. Ethyl-4-(((4-fluorophenyl)(hydroxy(phenoxy)phosphoryl)methyl)amino)benzoate **5a**

White powder (111.5 mg, 63%). ^1^H NMR (400 MHz, MeOD) δ(ppm): 1.28 (t, *J* = 7.1 Hz, 3H, **CH_3_**CH_2_O), 4.21 (q, *J* = 7.1 Hz, 2H, CH_3_**CH_2_**O), 4.70 (d, *J* = 23.4 Hz, 1H, **CH**-P), 6.50 (d, *J* = 8.8 Hz, 2H, H_arom_), 6.91–7.04 (m, 5H, H_arom_), 7.14–7.18 (m, 2H, H_arom_), 7.42–7.46 (m, 2H, H_arom_), 7.65 (d, *J* = 8.8 Hz, 2H, H_arom_), ^13^C NMR (100 MHz, MeOD) δ(ppm): 14.7, 56.6 (d, *J*_CP_ *J* = 145.1 Hz) 61.4, 113.2, 115.6 (d, *J =* 2.6 Hz), 115.8 (d, *J =* 2.2 Hz), 119.2, 122.0 (d, *J =* 4.0 Hz), 124.5, 130.2, 130.8 (d, *J =* 4.8 Hz), 130.9 (d, *J =* 5.0 Hz), 132.2, 135.7 (d, *J =* 2.9 Hz), 153.2 (d, *J =* 12.8 Hz), 154.0 (d, *J =* 7.8 Hz), 163.4 (d, *J_CF_* = 240.3 Hz), 168.8 (C=O). ^31^P NMR (162 MHz, MeOD) δ(ppm): 12.33. HRMS (ESI^+^) *m*/*z*, calcd. for C_22_H_22_FNO_5_P [M + H]^+^ 430.1220; found 430.1134.

#### 3.2.8. Ethyl-4-(((4chlorophenyl)(hydroxy(phenoxy)phosphoryl)methyl)amino)benzoate, **5b**

White powder (112.5 mg, 65%). ^1^H NMR (400 MHz, MeOD) δ(ppm): 1.28 (t, *J* = 7.1 Hz, 3H, **CH_3_**CH_2_O), 4.20 (q, *J* = 7.1 Hz, 2H, CH_3_**CH_2_**O), 4.70 (d, *J* = 23.6 Hz, 1H, **CH**-P), 6.50 (d, *J* = 8.8 Hz, 2H, H_arom_), 6.94–7.07 (m, 3H, H_arom_), 7.12–7.24 (m, 4H, H_arom_), 7.46 (dd, *J* = 8.5, 2.3 Hz, 2H, H_arom_), 7.70 (d, *J* = 8.8 Hz, 2H, H_arom_).^13^C NMR (100 MHz, MeOD) δ(ppm): 14.7, 56.9 (d, *J*_CP_ = 143.3 Hz), 61.4, 113.2, 119.2, 122.0 (d, *J =* 4.0 Hz), 124.5, 129.1 (d, *J =* 2.6 Hz), 130.2, 130.7 (d, *J =* 4.8 Hz), 132.2, 133.6 (d, *J =* 3.6 Hz), 138.7 (d, *J =* 3.0 Hz), 153.1 (d, *J =* 12.6 Hz), 154.0 (d, *J =* 8.1 Hz), 168.8 (C=O). ^31^P NMR (162 MHz, MeOD) δ(ppm): 12.16. HRMS (ESI^+^) *m*/*z*, calcd. for C_22_H_22_ClNO_5_P [M + H]^+^ 446.0924; found 446.0846.

#### 3.2.9. Ethyl-4-(((4-bromophenyl)(hydroxy(phenoxy)phosphoryl)methyl)amino)benzoate, **5c**

^1^H NMR (400 MHz, MeOD) δ(ppm): 1.29 (t, *J* = 7.2 Hz, 3H, **CH_3_**CH_2_O), 4.22 (q, *J* = 7.2 Hz, 2H, CH_3_**CH_2_**O), 4.68 (d, *J* = 23.6 Hz, 1H, **CH**-P), 6.50–6.55 (m, 3H, H_arom_), 7.01–7.05 (m, 1H, H_arom_), 7.16–7.20 (m, 1H, H_arom_), 7.35–7.40 (m, 5H, H_arom_), 7.66–7.68 (m, 3H, H_arom_).^13^C NMR (100 MHz, MeOD) δ(ppm): 14.7, 56.9 (d, *J*_CP_ = 139.2 Hz), 61.4, 113.3, 119.2, 122.0 (d, *J =* 4.0 Hz), 124.5, 130.2, 130.9 (d, *J =* 4.4 Hz), 131.1 (d, *J =* 5.2 Hz), 132.0 (d, *J =* 6.5 Hz), 132.1 (d, *J = 1*3.4 Hz),140.1(d, *J =* 3.0 Hz), 153.7 (d, *J =* 12.6 Hz), 154.8 (d, *J =* 8.1 Hz), 168.8 (C=O). ^31^P NMR (162 MHz, MeOD) δ(ppm): 14.51. HRMS (ESI^+^) *m*/*z*, calcd. for C_22_H_22_BrNO_5_P [M + H]^+^ 490.0419; found 490.0316.

#### 3.2.10. Ethy-l4-(((hydroxy(phenoxy)phosphoryl)(1-methyl-1H-pyrazol-4-yl)methyl)amino)benzoate, **5d**

White powder (225.4 mg, 54%). ^1^H NMR (400 MHz, DMSO_-_*d*_6_) δ(ppm): 1.24 (t, *J* = 7.1 Hz, 3H, **CH_3_**CH_2_O), 3.66 (s, 3H, **CH_3_-N**), 4.18 (q, *J* = 7.1Hz, 2H, CH_3_**CH_2_**O), 4.50 (dd, *J* =22.4, 9.3 Hz 1H, P**-CH-**NH), 6.49 (bs, 1H, H_arom_), 6.63 (d, *J* = 8.5 Hz, 2H, H_arom_), 6.92–6.96 (m, 1H, H_arom_), 7.05–7.07 (m, 2H, H_arom_), 7.14–7.18 (m, 2H, H_arom_), 7.35–7.41 (m, 1H, CH-**NH**-P), 7.59–7.61 (m, 3H, H_arom_).^13^C NMR (100 MHz, DMSO_-_*d*_6_) δ(ppm): 14.5, 38.3, 46.8 (d, *J*_CP_ = 149.2 Hz) 59.6, 111.7, 116.1, 119.4, 120.8 (d, *J =* 3.9 Hz), 122.2, 128.9, 129.8, 130.7, 138.4 (d, *J =* 4.8 Hz), 152.4 (d, *J =* 11.6 Hz), 153.6, 166.0 (C=O). ^31^P NMR (162 MHz, MeOD) δ(ppm): 10.13. HRMS (ESI^+^) *m*/*z*, calcd. for C_20_H_23_N_3_O_5_P [M + H]^+^ 416.1375; found 416.1337.

#### 3.2.11. Ethyl-4-(((hydroxy(phenoxy)phosphoryl)(3-phenyl-1H-pyrazol-4-yl)methyl)amino)benzoate, **5e**

White powder (282.2 mg, 49%).^1^H NMR (400 MHz, MeOD) δ(ppm): 1.20 (t, *J* = 7.1 Hz, 3H, **CH_3_**CH_2_O), 4.12 (q, *J* = 7.1 Hz, 2H, CH_3_**CH_2_**O), 4.77 (d, *J* = 21.7 Hz, 1H, **CH-P**), 6.13 (d, *J* = 8.4 Hz, 2H, H_arom_), 6.91–6.95 (m, 1H, H_arom_), 6.99–7.01 (m, 2H, H_arom_), 7.08–7.11 (m, 2H, H_arom_), 7.29–7.30 (m, 3H, H_arom_), 7.42 (d, *J* = 8.5 Hz, 2H, H_arom_), 7.52–7.54 (m, 2H, H_arom_), 7.82 (s, 1H, H_arom_). ^13^C NMR (100 MHz, MeOD) δ(ppm): 14.7, 46.6 (d, *J*_CP_ = 154.3 Hz), 61.3, 113.0, 117.2, 118.8, 122.1 (d, *J =* 4.0 Hz), 124.6, 129.4, 129.8 (d, *J =* 14.0 Hz), 130.2, 132.0, 152.8 (d, *J =* 11.6 Hz), 153.8 (d, *J =* 7.8 Hz), 168.7 (C=O). ^31^P NMR (162 MHz, MeOD) δ(ppm): 13.40. HRMS (ESI^+^) *m*/*z*, calcd. for C_25_H_25_N_3_O_5_P [M + H]^+^ 478.1532; found 478.1513.

#### 3.2.12. Ethyl-4-(((3-(4-fluorophenyl)-1H-pyrazol-4-yl)(hydroxy(phenoxy)phosphoryl)methyl)amino)benzoate, **5f**

White powder (354 mg, 61%). ^1^H NMR (400 MHz, MeOD) δ(ppm): 1.21 (t, *J* = 7.1 Hz, 3H, **CH_3_**CH_2_O), 4.13 (q, *J* = 7.1 Hz, 2H, CH_3_**CH_2_**O), 4.68 (d, *J* = 21.5 Hz,1H,**CH**-P), 6.14 (d, *J* = 8.6 Hz, 2H, H_arom_), 6.89–7.05 (m, 5H, H_arom_), 7.09–7.13 (m, 2H, H_arom_), 7.46 (d, *J* = 8.6 Hz, 2H, H_arom_), 7.52–7.64 (m, 2H, H_arom_), 7.87 (s, 1H, H_arom_). ^13^C NMR (100 MHz, MeOD) δ (ppm): 14.7, 46.7 (d, *J*_CP_ = 153.4 Hz), 61.3, 113.0, 116.4 (d, *J =* 21.7.0 Hz), 117.4, 118.8, 122.2 (d, *J =* 3.9 Hz), 124.6, 130.2, 131.9 (d, *J =* 8.3 Hz),132.0, 152.9 (d, *J =* 11.5 Hz), 153.9 (d, *J =* 7.8 Hz), 164.2 (d, *J_CF_ =* 246.5 Hz), 168.7 (C=O). ^31^P NMR (162 MHz, MeOD) δ(ppm): 13.42. HRMS (ESI^+^) *m*/*z* Calcd. for C_25_H_24_FN_3_O_5_P [M + H]^+^ 496.1438; found 496.1340.

### 3.3. In Vitro Antifungal Activity 

A total of 23 isolates of the genus *Scedosporium* were tested: *S. apiospermum* (5), *S. boydii* (5), *S. dehoogii* (5), *S. aurantiacum* (5) and *S. angustum* (3). All isolates were obtained from the Microbiology Department of the School of Medicine of Universidad Autónoma de Nuevo León and were previously identified by Elizondo-Zertuche et al. [10]. The strains were incubated on a potato dextrose agar (PDA) medium at 35 °C for their metabolic reactivation.

For the in vitro susceptibility studies, a preliminary test was carried out by the agar diffusion method against three strains of the *Scedosporium* species (ZS-025, 00-320, MC-070) versus the six α-aminophonates (**4a**–**f**) and six monohydrolyzed α-aminophosphonic acids (**5a**–**f**) previously synthesized, and in this way, the most active compound was selected based on its inhibition zone. For this, a grass-type seeding was carried out on Müller–Hinton medium (MCD Lab) with a concentration of 0.4 × 10^4^–5 × 10^4^ conidia/mL and disks impregnated with the 12 compounds at concentrations of 1–10 mg/mL were placed. The compound was determined as the one that presented a greater inhibition halo with respect to the control voriconazole (VRC). Subsequently, the most active compound, the monohydrolyzed α-aminophosphonic acid (**5f**), was evaluated at different concentrations using the liquid medium susceptibility method against the three *Scedosporium* strains mentioned previously (ZS-025, 00-320, MC-070). For this, in 5 mL snap tubes, 900 µL of nutrient broth (BD Bioxon) was added at a concentration of 0.4 × 10^4^–5 × 10^4^ conidia/mL, and then 100 µL of the compound were added at concentrations between 100–1000 µg/mL. The tubes were vortexed, incubated at 35 °C for 72 h and the concentration range was determined for subsequent evaluation by the CLSI method M38-A2.

Finally, the in vitro antifungal susceptibility of the 23 strains of *Scedosporium* species was determined by the macrodilution method for filamentous fungi according to the CLSI M38-A2 protocol. The antifungal agents used were amphotericin B (AMB), fluconazole (FLC) and voriconazole (VRC), which were obtained as pure reagent grade powders for AMB and VRC and as a medical solution for FLC. Final drug concentrations ranged from 0.125 to 64 μg/mL for FLC, 0.03 to 16 μg/mL for VRC and AMB and 500 to 700 μg/mL for compound **5f**. The tubes were incubated at 37 °C for 72 h and the minimal inhibitory concentration (MIC) for all antifungals was read visually. The MIC was defined as the drug concentration for which a ≥50% reduction in turbidity compared with the drug-free control for fluconazole or a 100% reduction in turbidity compared with the drug-free control for amphotericin B, voriconazole and **5f** compound was observed. Assays were done in duplicate using *Candida parapsilosis* ATCC 22019 and *Paecilomyces variotii* MYA 3630 (obtained from ATTC) as quality control organisms.

### 3.4. Evaluation of Cytotoxic Activity 

#### 3.4.1. Cell Culture 

The monkey kidney cell line (COS-7) used for biological tests was obtained from Centro Médico Siglo XXI.

The cell line was cultured in RPMI-1640 culture medium supplemented with fetal bovine serum (10%) and an antibiotic–antifungal mixture (1%).

#### 3.4.2. MTT Assay 

The two components that showed antifungal activity against *Scedosporium* spp. were evaluated by MTT assay to evaluate their cytotoxicity in healthy COS-7 cells. Briefly, cells were harvested with EDTA trypsin solution, already detached from the base of the culture flask, harvested and diluted with supplemented medium to inactivate trypsin. An aliquot of cells was taken to perform the viability count through the trypan blue technique using an electronic counter. The inoculum density was adjusted to 10 × 10^4^ cells/mL and they were deposited in a volume of 100 µL in 96-well plates. Cell cultures were incubated for 24 h to promote their adherence to the substrate at the bottom of the well. Next, the compounds were added in solution with the supplemented medium and their corresponding solvent in a volume of 100 µL. The compounds were prepared at a concentration of 40 mM in DMSO and/or ethanol; the final concentration was 1000 µM for the test drugs and ≤1% for DMSO. The etoposide was prepared at 20 mM in DMSO. At 24 h, the cell was removed from the medium, washed with a phosphate buffer and 100 µL of a MTT solution (3-(4, 5-dimethylthiazol-2-yl)-2,5-diphenyltetrazolium) was immediately added, after which they were left for 4 h in the incubator. Afterwards, the MTT solution was removed and 100 µL of DMSO were added to favor the solubility of formazan produced during cell metabolism; optical density (OD) was measured in a microplate reader at a wavelength of 470 nm.

Cell viability was calculated from the following expression: % Viability = (OD treatment/OD vehicle) × 100. Data are processed individually or by independent experiment, obtaining the average of these plus standard error of the mean.

### 3.5. Docking

The PyMOL 3.1 program was used for the preparation of the ligand and the protein and for the visualization of the molecular structure. The crystal structure of the protein was obtained from the Protein Data Bank (PDB) with the access code 5HS1. The molecules were optimized in the Avogrado program to obtain the structure with the lowest energy conformation; later, it was converted into the pdbqt format in order to perform the calculations. The calculations were carried out with AutoDock Vina. Briefly, AutoDock Vina is a software that performs a computerized docking study of the ligand with which it has a dihedral flexibility and a specific binding site of the protein, the program performing several simulations in each experiment. Each of these simulations shows a predictive mode of protein–ligand binding. In this experiment, water molecules and voriconazole (crystallized ligand) were removed from the protein file in pdbqt format. For validation of the docking protocol, the coordinations with the ligand were removed. The coordinates of the polar protein–ligand interactions were found from these coordinates and several simulations were run with different sizes of “box” (10 to 60 Å); with the help of the PyMOL program, the coordinate and the box size closest to a root mean square deviation (RMSD) of 2.5 were chosen. All calculations for protein-fixed ligand-flexible docking were analyzed using the Lamarckian genetic algorithm (LGA) method. The docking site on 14 α-demethylase was defined by establishing a grid box using PyMOL 3.1. The grid box size for the coordinates of *x*, *y* and *z* was 60 Å, with a grid spacing of 0.375 Å, centered on *x* = 106.102, *y* = 11.437 and *z* = 19.340 Å. The best conformation was chosen based on the lowest binding energy after the docking search was completed. In the AutoDock Vina configuration files, the parameter number modes were set to 1000 modes and exhaustiveness to 1000. 

With this information, the compounds were run in the AutoDock Vina program to evaluate the interaction energies of the compounds with the protein. The total number of runs was 1000. Finally, the interactions between the molecules and the amino acid residues of the protein were seen with the Discovery Studio Visualizer program.

## 4. Conclusions

New series of α-aminophosphonates and monohydrolyzed α-aminophosphonic acids were synthesized by simple, low cost, swift and non-conventional methodologies that comply with some green chemistry principles. Partial hydrolysis of α-aminophosphonates using potassium carbonate deserves special mention because of the advantages of using a weak base as a catalyst instead of harsh reaction conditions. The synthesized compounds were evaluated against 23 different strains of the *Scedosporium* species. Being the first report of derivatives of aminophosphonates tested against the *Scedosporium* genus, it was found that the compound **5f** shows activity against this species at concentrations of 645–700 µm/mL. Also, this compound was less toxic than voriconazole in COS-7 cells at equivalent concentrations, so **5f** represents a molecule with attractive properties in its current form, which could be enhanced with some modifications. It is a viable and promising compound to be evaluated through in vitro and in vivo studies against a wide variety of filamentous fungi of medical importance.

## Figures and Tables

**Table 1:** Reaction time and yields for the synthesis of compounds **4a**–**f.**

Compound	Time (min)	Yield (%)
**4a**	15	77
**4b**	25	65
**4c**	5	50
**4d**	15	92
**4e**	15	98
**4f**	15	95

**Table 2:** ^1^H and ^13^C-NMR spectroscopic data for *J* coupling CH-P, C-P and C-F in compounds **4a**–**f** and **5a**–**f**.

Compound	^1^H NMR *J* Coupling CH-P (Hz)	^13^C NMR *J* Coupling C-P (Hz)	^13^C NMR *J* Coupling C-F (Hz)
**4a**	24.9	156.2	244.3
**4b**	25.1	156.2	-
**4c**	25.4	156.4	-
**4d**	22.1	163.0 [36]	-
**4e**	18.8	165.1	-
**4f**	19.9	164.7	248.1
**5a**	23.4	145.1	240.3
**5b**	23.6	143.3	-
**5c**	23.6	139.2	-
**5d**	22.4	149.2	-
**5e**	21.7	154.3	-
**5f**	21.5	153.4	246.5

**Table 3:** In vitro susceptibilities of 3 strains of *Scedosporium* against voriconazole antifungal and compounds **4a**–**f** and **5a**–**f** in the agar diffusion method.

Species /Antifungal	Range (mg/mL)	Range Inhibition Halo (mm/72 h)
*S. apiospermum* **(00-320)**		
VRC	0.016	≥10 mm
**4a**–**f**	1–10	1–≥1 mm
**5a**–**f**	1–10	2–≥10 mm
**5f**	0.1–1	7–≥10 mm
*S. boydii* **(ZS-025)**		
VRC	0.016	≥10 mm
**4a**–**f**	1–10	1–≥1 mm
**5a**–**f**	1–10	1–≥9 mm
**5f**	0.1–1	5–≥10 mm
*S. aurantiacum* **(MC-070)**		
VRC	0.016	≥10 mm
**4a**–**f**	1–10	1–≥1 mm
**5a**–**f**	1–10	2–≥9 mm
**5f**	0.1–1	3–≥10 mm

VRC (control), voriconazole.

**Table 4:** In vitro susceptibilities of 23 strains of *Scedosporium* against antifungal drugs and compound **5f**.

Species (n) /Antifungal	MIC_100_ Range (µg/mL)	MIC_100_ GM (µg/mL)
***S. apiospermum* (5) **		
AMB	2–16	6. 96
VRC	1–4	2.64
FLC	64–>64	64
**5f**	640–685	648. 76
***S. aurantiacum* (5)**		
AMB	16–>16	16
VRC	0.5–1	0.76
FLC	32–64	55.71
**5f**	700–>700	700
***S. dehoogii*** **(5)**		
AMB	16–>16	16
VRC	0.5–1	0.87
FLC	32–64	36.75
**5f**	700–>700	700
***S. boydii*** **(5)**		
AMB	8–>16	8
VRC	0.25–1	0.44
FLC	32–>64	45.25
**5f**	610–>700	672.20
***S. angustum*** **(3)**		
AMB	8–>16	8
VRC	0.5–1	0.63
FLC	32–64	50.80
**5f**	700	700

AMB, amphotericin B; FLC, fluconazole; VRC, voriconazole; GM, geometric mean.

**Table 5:** Cytotoxicity in a healthy COS-7 cell against voriconazole antifungal and compounds **5a** and **5f** explored by MTT assay.

Antifungal/Compounds (1000 μM)	% Viability
VRC	65.80 ± 1.79
**5a**	55.20 ± 1.79
**5f**	73.51 ± 4.88
Etoposide *	84.0 ± 2.0

* Control (25 μM), VRC, Voriconazole.

## Sample Availability

Samples of the compounds are not available from the authors.
